# Slot Blot Analysis of Intracellular Glyceraldehyde-Derived Advanced Glycation End Products Using a Novel Lysis Buffer and Polyvinylidene Difluoride Membrane

**DOI:** 10.21769/BioProtoc.5038

**Published:** 2024-07-20

**Authors:** Takanobu Takata, Hiroki Murayama, Togen Masauji

**Affiliations:** 1 Department of Life Science, Medical Research Institute, Kanazawa Medical University, Uchinada, Japan; 2 Department of Pharmacy, Kanazawa Medical University Hospital, Uchinada, Japan

**Keywords:** Advanced glycation end product, Glyceraldehyde, GA-AGE, Polyvinylidene difluoride, 2-amino-2-hydromethyl-1, 3-propanediol, Urea, Thiourea, 3-[(3-cholamidopropyl)-dimethylammonio]-1-propanesulfonate, Slot blot analysis

## Abstract

Advanced glycation end products (AGEs) are formed through the reaction/modification of proteins by saccharides (e.g., glucose and fructose) and their intermediate/non-enzymatic products [e.g., methylglyoxal and glyceraldehyde (GA)]. In 2017, Dr. Takanobu Takata et al. developed the novel slot blot method to quantify intracellular GA-derived AGEs (GA-AGEs). Although the original method required nitrocellulose membranes, we hypothesized that the modified proteins contained in the AGEs may be effectively probed on polyvinylidene difluoride (PVDF) membranes. Because commercial lysis buffers are unsuitable for this purpose, Dr. Takata developed the slot blot method using an in-house-prepared lysis buffer containing 2-amino-2-hydromethyl-1,3-propanediol (Tris), urea, thiourea, and 3-[(3-cholamidopropyl)-dimethylammonio]-1-propanesulfonate (CHAPS) that effectively probes AGEs onto PVDF membranes. The slot blot method also entails the calculation of Tris, urea, thiourea, and CHAPS concentrations, as well as protein and mass to be probed onto the PVDF membranes. GA-AGE-modified bovine serum albumin (BSA, GA-AGEs-BSA) is used to draw a standard curve and perform neutralization against a non-specific combination of anti-GA-AGEs antibodies, thereby enabling the quantification of GA-AGEs in cell lysates. This paper presents the detailed protocol for slot blot analysis of intracellular GA-AGE levels in C2C12 cells.

Key features

• This protocol leverages the idea that advanced glycation end products are modified proteins.

• The lysis buffer containing Tris, urea, thiourea, and CHAPS enables probing proteins onto PVDF membranes.

• Intracellular GA-AGE levels may be quantified for some cell types using polyclonal anti-GA-AGE antibodies and standard GA-AGE-modified BSA.

• The lysis buffer may be simultaneously prepared with the cell lysate.

• There is no limit to the type of cultured cells used in the preparation of cell lysate.

## Graphical overview


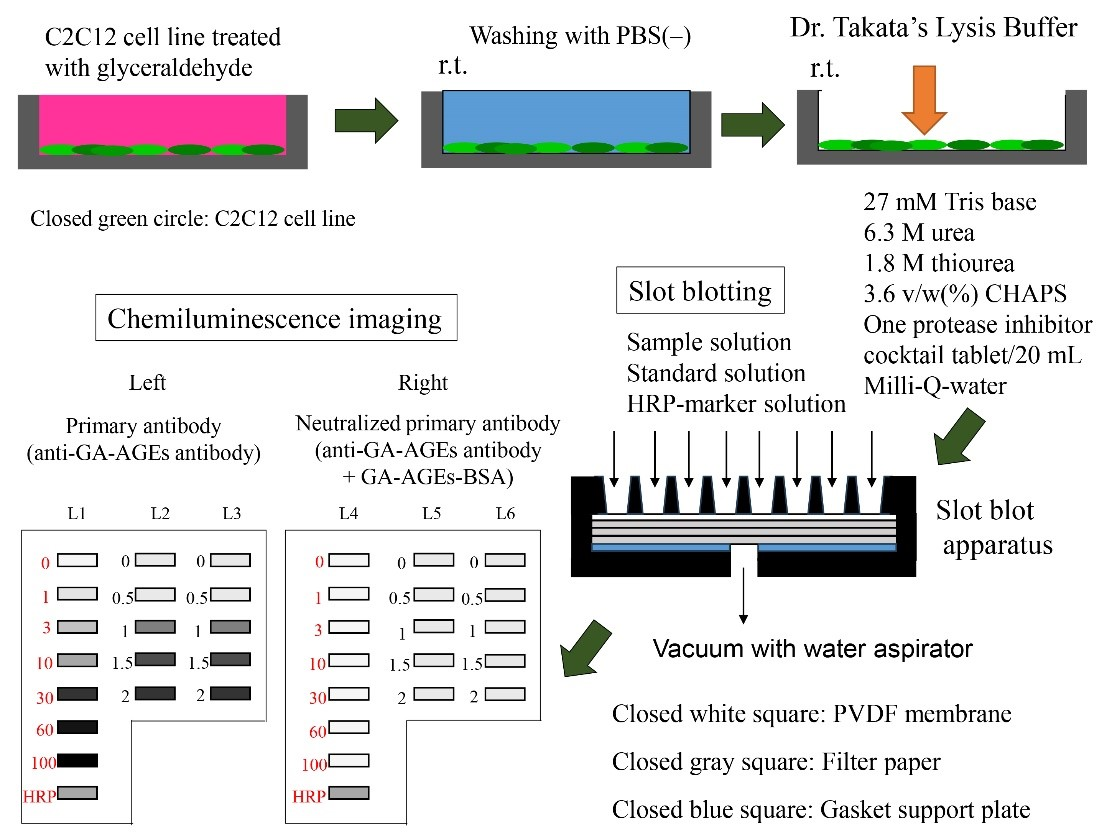


**Preparation of sample and slot blot analysis with Dr. Takata’s lysis buffer and polyvinylidene difluoride (PVDF) membranes.**

## Background

The quantification of intracellular advanced glycation end products (AGEs) is particularly useful in the fields of biochemistry, molecular biology, and protein engineering [1]. AGEs are a type of modified proteins that can be extracted from cultured cells and tissues [2] and indirectly measured using the enzyme-linked immunosorbent assay (ELISA) [3,4]. However, some researchers prefer using a slot blot approach [5–7], because identifying or quantifying some types of AGEs may be difficult using ELISA. Previous slot blot methods for the quantification of AGEs were limited by their use of nitrocellulose membranes and radioimmunoprecipitation (RIPA) buffer [5,6]. Although nitrocellulose membranes are useful in column chromatography, polyvinylidene difluoride (PVDF) membranes are more durable and show greater protein adsorption ability [8,9]. However, there is a lack of suitable lysis buffers for probing proteins onto PVDF membranes. Although RIPA buffer is used in western blotting, it is less suitable in slot blot analyses with PVDF membranes [8,9]. Consequently, Dr. Takanobu Takata developed a lysis buffer containing 2-amino-2-hydromethyl-1,3-propanediol (Tris), urea, thiourea, and 3-[(3-cholamidopropyl)-dimethylammonio]-1-propanesulfonate (CHAPS) to quantify intracellular AGEs using the slot blot analysis [8]. The improved probing efficacy of this lysis buffer may be related to protein carbamoylation [8,9] and the absence of Triton-X, which can inhibit the probing of proteins onto PVDF membranes [8,9]. In 2017, the novel slot blot method was used to accurately quantify intracellular glyceraldehyde (GA)-derived AGEs (GA-AGEs) [10]. From 2017 to 2022, Takata et al. applied this method in the quantification of intracellular GA-AGEs in cells and tissue lysates of the pancreas [10,11], heart [12,13], skeletal muscles [14], liver [15–19], and bone [20] using (i) the novel lysis buffer, (ii) standard GA-AGEs modified bovine serum albumin (BSA), and (iii) neutralization using anti-GA-AGEs antibodies. In this method, the standard GA-AGEs-BSA is used to estimate standard curves [10]; neutralization using a non-specific combination of polyclonal anti-GA-AGEs antibodies [21] avoids biases in the quantification of GA-AGEs [8, 10–17]. We previously applied this protocol to a type of GA-AGEs [10–17] known as toxic AGEs (TAGE) [21]. However, our slot blot may be applied to various types of AGEs, including 1,5-anhydro-fructose AGEs [22] and modified proteins [8,9], even though various AGE modifications may occur in one protein type or molecule [23]. This study presents a validated protocol for the slot blot analysis of intracellular GA-AGE levels using Dr. Takata’s lysis buffer and a PVDF membrane.

## Materials and reagents

**Biological materials**

1. C2C12 cell line (KAC, Kyoto, catalog number: EC91031101-F0)
  The C2C12 cell line is an immortalized mouse myoblast cell line (ATCC, catalog number: CRL1772)

**Reagents**

1. Dulbecco’s modified Eagle’s medium (DMEM) (Sigma-Aldrich, catalog number: D6046-500M)
2. Penicillin/streptomycin solution (FUJIFILM Wako Pure Chemical Corporation, catalog number: 168-23191)
3. Fetal bovine serum (FUJIFILM Wako Pure Chemical, catalog number: 554-04855)
4. Glyceraldehyde (Nacalai Tesque, catalog number: 17014-81)
5. Phosphate-buffered saline (PBS) without Ca^2+^ and Mg^2+^ [PBS(-)], 20× (LSI Medience, catalog number: PM102-PN)
6. 2-amino-2-hydromethyl-1,3-propanediol (Tris base) (Tris) (FUJIFILM Wako Pure Chemical, catalog number: 011-20095)
7. Urea (FUJIFILM Wako Pure Chemical, catalog number: 217-01215)
8. Thiourea (FUJIFILM Wako Pure Chemical, catalog number: 206-17355)
9. CHAPS (DOJINDO, Kumamoto, Japan, catalog number: 349-04722)
10. Methanol (FUJIFILM Wako Pure Chemical, catalog number: 131-01826)
11. Protease Inhibitor cocktail cOmplete Tablets EDTA-free, EASY pack (Roche, catalog number: 04-693-132-001)
12. BSA fraction IV (FUJIFILM Wako Pure Chemical, catalog number: 019-2329)
13. Bradford dye reagent (Takara Bio, catalog number: T9310A-1)
14. Skim milk for immunoassay (Nacalai Tesque, catalog number: 31149-75)
15. Tween-20 (GE Healthcare, catalog number: 17-1316-01)
16. Polyclonal anti-GA-AGE antibodies (purchased from Prof. Masayoshi Takeuchi, Department of Advanced Medicine, Medical Research Institute, Kanazawa Medical University, Uchinada, Japan; 920-0293).
*Note: Prof. Takeuchi successfully prepared the polyclonal antibody for TAGE, despite having limited information on the structure of TAGE [21]. Takeuchi et al. reported the hypothetical structure of TAGE in 2023 [24]. The antibody is preserved at -80 °C.*
17. GA-AGE-BSA, 10 mg/mL (or TAGE-BSA, purchased from Prof. Takeuchi)
*Note: Despite limited information on the intra- and intermolecular structure of TAGE-BSA, the polyclonal anti-GA-AGE antibody (Reagent 17) developed by Prof. Takeuchi could be probed against the antigen recognition site in TAGE-BSA [21]. The GA-AGEs-BSA was dissolved in PBS and preserved at -30 °C.*
18. Horseradish peroxidase (HRP)-conjugated molecular weight marker (Bionexus, catalog number: BNPM41)
19. Polyclonal goat anti-rabbit immunological HRP-conjugated antibody (DAKO, catalog number: REF0448)
20. ImmunoStar LD kit (FUJIFILM Wako Pure Chemical, catalog number: 292-69903)
21. Milli-Q ultrapure water
*Note: Purchased with an RFU554CA ultrapure water system (Advantech Toyo, Tokyo, Japan) at the Kanazawa Medical University.*

**Solutions**

1. Medium for cell culture (see Recipes)
2. PBS(-) 1× (see Recipes)
3. Solution A (see Recipes)
4. Solution B (see Recipes)
5. Solution C (see Recipes)
6. Solution D (see Recipes)
7. 20 mg/mL BSA in Solution D (20 mg/mL BSA solution) (see Recipes).
8. Diluted BSA solution (see Recipes).
9. Diluted GA-AGEs-BSA solution (see Recipes)
10. HRP-conjugated molecular weight marker solution (see Recipes)
11. PBS-T (see Recipes)
12. 5% SM-PBS-T (see Recipes)
13. 0.5% SM-PBS-T (see Recipes)
14. Primary antibody solution (see Recipes)
15. Neutralized primary antibody solution (see Recipes)
16. Secondary antibody solution (see Recipes)

**Recipes**

1. **Medium for cell incubation**
  Mix the reagents on a laminar flow hood.
*Note: Because this step can easily be performed in-house prior to the experiment, we do not provide a detailed description here. Complete information may be found in our previous studies [10–16]. Media can be stored in the general refrigerator (4 °C).*
  **Table:** 

Reagent	Volume	Recommended storage condition
DMEM	450 mL	4 °C
Penicillin/streptomycin solution	5 mL	-30 °C
Fetal bovine serum	50 mL	-30 °C
Total	505 mL
2. **PBS(-) (1×)**
  Room temperature refers to 22–28 °C.
  **Table:** 

Reagent	Volume	Recommended storage condition
PBS(-) (20×)	50 mL	Room temperature
Milli-Q water	950 mL	Room temperature
Total	1,000 mL
3. **Solution A**
  Using a 50 mL polypropylene centrifuge tube and serological pipet, dissolve Tris in Milli-Q water (10 mL) to prepare 1 mol/L (M) Tris solution. Solution A may be stored at -30 °C for 6 months.
*Note: The pH of the Tris solution was approximately 9.4. Only Tris powder was dissolved in Milli-Q-water, without adding other reagents (e.g., hydrochloride).*
  **Table:** 

Reagent	Quantity/volume	Recommended storage condition	Final concentration
Tris	1.21 g	Room temperature	1 mol/L (M)
Milli-Q water	10 mL	Room temperature	
Total	10 mL
4. **Solution B**
  Dissolve one tablet of protease inhibitor cocktail (cOmplete Tablets EDTA free) in Milli-Q water (2 mL) within a microcentrifuge tube; transfer the Milli-Q water to the tube using a 100–1,000 μL Gilson PIPETMAN. We recommend Solution B (2 mL) to be prepared and used immediately with every experiment. However, Solution B can be preserved at -30 °C for three months if it is reused. Approximately 100 μL of Solution B were preserved.
  **Table:** 

Reagent	Quantity/Volume	Recommended storage condition	Final concentration
Protease inhibitor cocktail	1 tablet	4 °C	1 tablet/2 mL
Milli-Q water	2 mL	Room temperature	
Total	2 mL
5. **Solution C**
  Solution C contains 30 mM Tris, 7 M urea, 2 M thiourea, and 4% CHAPS. Mix these reagents in a 50 mL polypropylene centrifuge tube and add Milli-Q water to a final volume of 20 mL. Treat 1 M Tris with Milli-Q water using a 200–1,000 μL Gilson PIPETMAN.
*Note: The reagents are added to the centrifuge tube in no specific order. At room temperature, urea and thiourea are not easily dissolved in Milli-Q water; accordingly, we recommend using a vortex system (e.g., Vortex-Genie 2) at room temperature. We do not recommend dissolution in a 37 °C CO_2_ incubator or water bath because urea and thiourea may produce cyanate or isocyanic acid at high temperatures [9]. Solution C can be preserved at -30 °C for three months, though we recommend preparing it immediately before use.*
  **Table:** 

Reagent	Quantity/Volume	Recommended storage condition	Final concentration
1 M Tris	0.6 mL	Room temperature	30 mM
Urea	8.40 g	Room temperature	7 M
Thiourea	3.04 g	Room temperature	2 M
CHAPS	0.80 g	Room temperature	4 w/v (%)
Milli-Q water	20 mL	Room temperature	
Total	20 mL
6. **Solution D**
  Prepare Solution D by mixing Solutions B and C at a ratio of 1:9. Transfer Solution C using the 200–1,000 μL Gilson PIPETMAN. Transfer Solution D using a 10 mL serological pipette. Solution D contains 27 mM Tris, 6.3 M urea, 1.8 M thiourea, and 3.6 v/w (%) CHAPS.
*Note: We recommend dissolution using a vortex system (e.g., Vortex-Genie 2) at room temperature. Solution D can be preserved at -30 °C for three months, though we recommended preparing it immediately before use. Solution D was used as lysis buffer to prepare the cell lysate [8–17], which was prepared from C2C12 cells treated with glyceraldehyde [14]. Although “Modified Solution C” containing 30 mM Tris, 7 M urea, 2 M thiourea, 4% CHAPS, and 4% Solution B may also be used as lysis buffer [18–20,22], we recommend that Solution D be used because it contains sufficient urea, thiourea, and CHAPS to generate the carbamylated AGE proteins [8,9]. The cell lysates and unused Solution D should be preserved at -30 °C until later use in the slot blot experiment. The condition of the unused Solution D and the cell lysates should be arranged.*
  **Table:** 

Reagent	Volume	Final concentration
Solution B	2 mL	10%
Solution C	18 mL	90%
Total (optional)	20 mL
7. **BSA–Solution D mixture (20 mg/mL BSA solution)**
  Dissolve 20 mg of BSA in Solution D (10 mL) within a 50 mL polypropylene centrifuge tube. Transfer Solution D using a 200–1,000 μL Gilson PIPETMAN.
*Note: BSA cannot be easily dissolved in Solution D; thus, we recommend using a vortex system (e.g., Vortex-Genie 2) at room temperature. The presence of undissolved BSA can be confirmed by the presence of a gelatinous substance. Dissolution by vortex should not be performed at high temperatures (e.g., 37 °C) [9].*
*Note: We do not recommend commercial BSA for use in this experiment. An in-house solution should preferably be prepared. We believe that BSA fraction IV is suitable for the Bradford method in our protocol.*
  **Table:** 

Reagent	Quantity/Volume	Final concentration	Recommended storage condition
BSA	20 mg	2.0 mg/mL (μg/μL)	4 °C
Milli-Q water	10 mL		Room temperature
Total	10 mL
8. **Diluted BSA solution**
  Dilute 20 mg/mL BSA solution to 0.0625, 0.125, 0.25, 0.5, 1.0, and 1.5 μg/μL in a 1.5 mL microcentrifuge tube; transfer BSA using a 50–200 μL Gilson PIPETMAN. We recommend that dissolution be performed using a vortex system (e.g., Vortex-Genie 2) at room temperature. The BSA solution should be made and used immediately with every experiment. BSA solution should be aliquoted (approximately 100 μL) and preserved at -80 °C if it will be reused. However, we recommend preparing and using a fresh BSA solution per experiment.
*Note: Prepare from 20 mg/mL BSA solution (Recipe 7).*
  **Table:** 

Volume of reagents	Volume of Solution D	Total volume	Final concentration
150 μL of 2.0 μg/μL BSA solution	50 μL	200 μL	1.5 μg/μL
100 μL of 2.0 μg/μL BSA solution	100 μL	200 μL	1.0 μg/μL
100 μL of 1.0 μg/μL BSA solution	100 μL	200 μL	0.5 μg/μL
100 μL of 0.5 μg/μL BSA solution	100 μL	200 μL	0.25 μg/μL
100 μL of 0.25 μg/μL BSA solution	100 μL	200 μL	0.125 μg/μL
100 μL of 0.125 μg/μL BSA solution	100 μL	200 μL	0.0625 μg/μL
9. **Diluted GA-AGEs-BSA solution**
  Dilute 10 mg/mL GA-AGEs-BSA in PBS(-) within a 1.5 mL microcentrifuge tube; transfer the solution using a 50–200 μL or 1–10 μL Gilson PIPETMAN.
*Note: We recommend the diluted GA-AGEs-BSA solution to be prepared before being used.*
  **Table:** 

Volume of reagents	Volume of PBS(-)	Total volume	Final concentration
2.0 μL of 10 mg/mL GA-AGEs-BSA	98 μL	100 μL	200 ng/μL
20 μL of 200 ng/μL GA-AGEs-BSA	60 μL	80 μL	50 ng/μL
60 μL of 50 ng/μL GA-AGEs-BSA	40 μL	100 μL	30 ng/μL
30 μL of 50 ng/μL GA-AGEs-BSA	70 μL	100 μL	15 ng/μL
10 μL of 50 ng/μL GA-AGEs-BSA	90 μL	100 μL	5 ng/μL
10 μL of 15 ng/μL GA-AGEs-BSA	90 μL	100 μL	1.5 ng/μL
10 μL of 5 ng/μL GA-AGEs-BSA	90 μL	100 μL	0.5 ng/μL
10. **HRP-conjugated molecular weight marker solution**
  Dilute the HRP-conjugated molecular weight marker in PBS(-) within a 1.5 mL microcentrifuge tube; transfer the solution using the 200–1,000 μL or 1–10 μL Gilson PIPETMAN.
*Note: We recommend dissolution to be performed using a vortex system (e.g., Vortex-Genie 2) at room temperature and the diluted GA-AGEs-BSA solution to be prepared at the time of use.*
  **Table:** 

Reagent	Volume	Recommended storage condition
HRP-conjugated molecular weight marker	3 μL	-30 °C
PBS(-)	197 μL	Room temperature
Total	200 μL
11. **PBS-T**
  Dissolve Tween-20 in PBS(-). Transfer Tween-20 using the 200–1,000 μL Gilson PIPETMAN.
  **Table:** 

Reagent	Volume	Recommended storage condition
Tween-20	0.5 mL	Room temperature
PBS(-)	1,000 mL	Room temperature
Total	1,000.5 mL
12. **5% SM-PBS-T for immunoassay**
  Dissolve skim milk for immunoassay in PBS-T within a 50 mL polypropylene centrifuge tube; transfer PBS-T using the 10 mL serological pipette.
*Note: We recommend that dissolution be performed using a vortex system (e.g., Vortex-Genie 2) at room temperature and that 5% SM-PBS-T be prepared before use. However, the solution may be preserved at 4 °C for two days.*
  **Table:** 

Reagent	Quantity/Volume	Recommended storage condition
Skim milk for immunoassay	2.5 g	4 °C
PBS-T	50 mL	Room temperature
Total	50 mL
13. **0.5% SM-PBS-T**
  Dilute 5% SM-PBS-T to 0.5% SM-PBS-T in a 50 mL polypropylene centrifuge tube; transfer PBS-T using the 10 mL serological pipette.
*Note: We recommend that dissolution be performed using a vortex system (e.g., Vortex-Genie 2) at room temperature and that 5% SM-PBS-T be prepared before use, which may occur at room temperature. However, the solution may be preserved at 4 °C for two days.*
  **Table:** 

Reagent	Volume	Recommended storage condition
5% SM-PBS-T	5 mL	Room temperature
PBS-T	45 mL	Room temperature
Total	50 mL
14. **Primary antibody solution**
  Mix 5 mL of 0.5% SM-PBS-T (Recipe 13) using a 10 mL serological pipette with 5 μL of polyclonal anti-GA-AGE antibody (using a 2–20 μL Gilson PIPETMAN) within a 15 mL polypropylene centrifuge tube.
*Note: We recommend that dissolution be performed using a vortex system (e.g., Vortex-Genie 2) for 10 s at room temperature. The polyclonal anti-GA-AGE antibody, which was preserved at -80 °C, should first be defrosted at 0 °C (in ice) and may then be preserved at 4 °C for half a year.*
  **Table:** 

Reagent	Volume	Recommended storage condition	Dilution ratio
Polyclonal anti-GA-AGEs antibody	5 μL	-80 °C	1:1,000
0.5% SM-PBS-T	5.0 mL (5,000 μL)	Room temperature	
Total (optional)	5.005 mL (5,005 μL)
15. **Neutralized primary antibody solution**
  Mix 5 μL of polyclonal anti-GA-AGEs antibody (2–20 μL Gilson PIPETMAN) with 125 μL of 10 mg/mL GA-AGEs-BSA (50–200 μL Gilson PIPETMAN) and 4.875 mL of 0.5% SM-PBS-T (10 mL serological pipette) within a 15 mL polypropylene centrifuge tube.
*Note: We recommend that dissolution be performed using a vortex system (e.g., Vortex-Genie 2) for 10 s at room temperature. Polyclonal anti-GA-AGEs and GA-AGEs-BSA were preserved at -80 °C and -30 °C, respectively, and defrosted on ice. We recommend that 10 mg/mL GA-AGEs-BSA be divided into 130 μL aliquots and preserved at -30 °C in advance. Moreover, we recommended the divided and preserved GA-AGEs-BSA to be used, and one of them should be for one experiment (they should not be refrozen and reused).*
  **Table:** 

Reagent	Volume	Recommended storage condition	Dilution ratio/Final concentration
Polyclonal anti-GA-AGEs antibody	5 μL	-80 °C	1:1,000
10 mg/mL GA-AGEs-BSA	125 μL	-30 °C	1/40 (250 μg/mL)
0.5% SM-PBS-T	4.875 mL (4,875 μL)	Room temperature	
Total	5.005 mL (5,005 μL)
16. **Secondary antibody solution**
  Mix 5 mL of 0.5% SM-PBS-T (10 mL serological pipette) with 2.5 μL of polyclonal goat anti-rabbit immunological HRP-conjugated antibody (1–10 μL Gilson PIPETMAN) within a 15 mL polypropylene centrifuge tube. We recommend that dissolution be performed using a vortex system (e.g., Vortex-Genie 2) for 10 s at room temperature.
  **Table:** 

Reagent	Volume	Recommended storage condition	Dilution ratio
Polyclonal goat anti-rabbit immunological HRP-conjugated antibody	2.5 μL	4 °C	1:2,000
0.5% SM-PBS-T	5 mL (5000 μL)	Room temperature	
Total	5.0025 mL (5002.5 μL)

**Laboratory supplies**

1. 60 mm dish (BM Equipment, catalog number: 93060)
2. 1.5 mL microcentrifuge tube (Thermo Fisher Scientific, catalog number: 3451)
3. 2.0 mL microcentrifuge tube (Watson, catalog number: 132-6201)
4. 50 mL polypropylene centrifuge tube (TrueLine; Nippon Genetics, catalog number: TR2004)
5. 15 mL polypropylene centrifuge tube (TrueLine; Nippon Genetics, catalog number: TR2000)
6. 10 mL serological pipette (BM Equipment, catalog number: 207500-SLP-10)
7. Rubber bulb for 10 mL serological pipette (AS ONE, catalog number: 6-356-4)
8. Disposable polypropylene tray (AS ONE, catalog number: 1-3145-03)
9. Dispenser (TPP, BM Equipment, catalog number: 99010)
10. 1–10 μL pre-sterilized tip (10 μL) (BM Equipment, catalog number: W10-RS)
11. 50–200 μL pre-sterilized tip (200 μL) (Watson, catalog number: 62-0887-34)
12. 200–1,000 μL pre-sterilized tip (1,000 μL) (Watson, catalog number: 38688239)
13. 96-well microplates (Becton Dickinson, catalog number: REF353072)
14. 25 mL reagent reservoir (BM Equipment, catalog number: BM-0850-1)
15. PVDF membrane (pore size: 0.45 μm) (Merck Millipore, catalog number: IPVH00010)
16. Filter paper (9 cm × 12 cm) (Bio-Rad Laboratories, catalog number: 1620161)
17. Hybri-Bag (hard type) (Cosmo Bio, catalog number: S1001)
18. Gilson PIPETMAN (GILSON, model type: 100–1,000 μL)
19. Gilson PIPETMAN (GILSON, model type: 50–200 μL)
20. Gilson PIPETMAN (GILSON, model type: 2–20 μL)
21. Gilson PIPETMAN (GILSON, model type: 1–10 μL)
22. Nichipet 7000 range: 50–200 μL (Nichiryo, Tokyo, Japan)

## Equipment

1. Ultrapure Water System (Advance Toyo, model: RFU554CA)
2. Flask-trap aspirator (1,000 mL) (Biosan, Riza, Latvia; model number: FTA-1)
3. Centrifuge (Eppendorf, model: 5415R)
4. Microplate reader (Bio-Rad, model: iMark)
5. Vortex-Genie 2 (M&S Instruments, catalog number: 33230217)
6. Imager (M&S Instruments, model: Fusion FX)
7. AS-200 Heat sealer (AS ONE, catalog number: H221049/01167C)
8. Seesaw shaker (BIO CRAFT, Tokyo, model: BC-700)
9. Aspirator with water pump (AS ONE, catalog number: 1-689-02)
10. Bio-Dot SF microfiltration apparatus (48 lanes) (Bio-Rad; Figure 1)
  **Figure 1.**
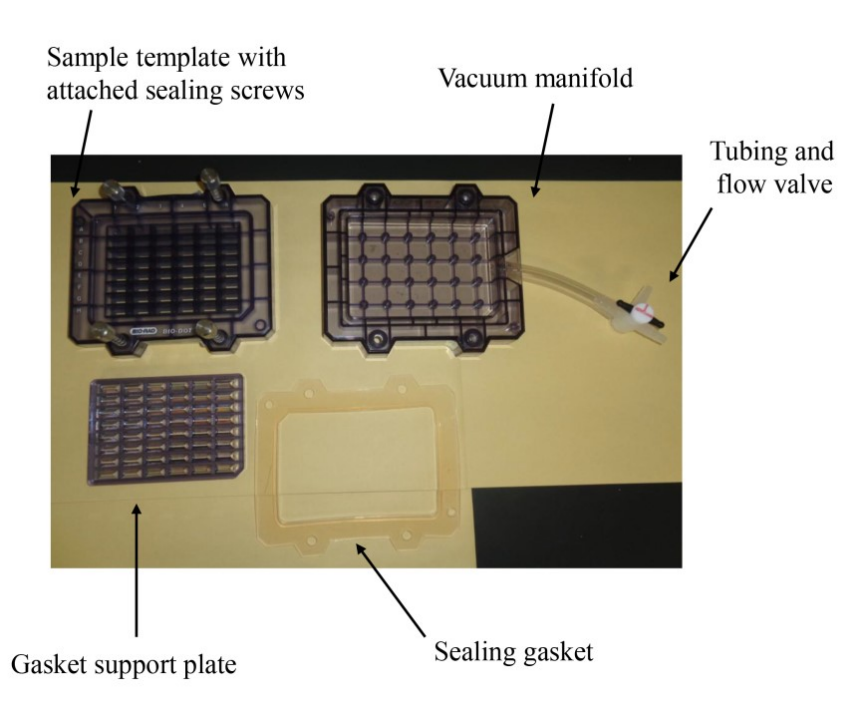
Bio-Dot SF microfiltration apparatus (48 lanes) containing sample template and equipped with sealing screws, sealing gasket, gasket support plate, vacuum manifold, and tubing with flow valve

## Software and datasets

1. Excel software (Microsoft, Redmond, WA, USA. version 2010, 2013, 2016)
*Note: The software was installed on a standard personal computer (OS: Windows 7, 10).*
2. FUSION FX software (M&S Instruments, version: 17.03)
*Note: The software was installed on a standard personal computer (OS: Windows 7) with Fusion FX imager (M&S Instruments) and was used since November 2017 at Kanazawa Medical University [11,13–16,22]. However, other chemiluminescence imagers and software may be used [10,12,17–20].*
3. Stat FX software (Artech, Osaka, Japan, version: 6)
*Note: When the slot blot analysis was performed in more than three independent experiments, Statflex (version 6) was used to perform statistical analysis [10–16,18–20].*

## Procedure

- **Incubation of C2C12 cell line and treatment with glyceraldehyde**
*Note: Here, we only provide the simplified procedure for the slot blot analysis. A detailed description may be found in the Materials and Methods in Takata et al. [14] (DOI: 10.1186/s13098-020-00561-z).*
  1. Seed 1.9 × 10^4^ cells/cm^2^ onto a 60 mm dish and incubate in a CO_2_ incubator for 24 h on DMEM supplemented with penicillin/streptomycin and fetal bovine serum.
  2. After changing the medium, treat the cells with 0, 0.5, 1, 1.5, and 2 mM glyceraldehyde and incubate in the CO_2_ incubator for 24 h.
*Note: The medium should be refreshed 24 h after seeding.*
- **Removing medium and washing cells with PBS(-)**
  1. Remove culture medium by decantation and aspirate the residual medium with a 1,000 mL trap-flask aspirator. Perform this step twice.
*Note: After decantation, ~300 μL of the culture medium should remain.*
  2. Add 7.0 mL of PBS(-), decant, and aspirate the residual PBS(-) with the flask-trap aspirator. Perform this step twice.
*Note: Transfer PBS(-) using a 10 mL serological pipette at 22–28 °C Decantation removes ~300 μL of PBS(-).*
- **Preparation of cell lysates**
  1. Add 300 μL of Solution D into a 60 mm dish and scrape and move the cells into a 1.5 mL microcentrifuge tube.
  2. Add cells to Solution D and harvest with a dispenser.
  3. Incubate cells in Solution D on ice for 20 min, during which the cell suspension is subjected to five pipetting operations, repeated three times.
*Note: This operation is performed with 5–6 min intervals.*
  4. Centrifuge cells at 10,000× *g* for 15 min at 4 °C with the 5415R centrifuge.
  5. Collect the supernatants in the 1.5 mL microcentrifuge tube.
*Note: Cell lysates are preserved at -80 °C. Both cell lysates and Solution D are preserved at -80 °C until the protein concentrations are measured, because the components of Solution D should remain in the same conditions.*
- **Measurement of protein concentration in cell lysates (see General note 1)**
  1. Add 4 μL of BSA in Solution D (final 0–2.0 μg/μL) to a 96-well microplate (N = 2).
  2. Add 4 μL of cell lysate (cells treated with 0, 0.5, 1, 1.5, and 2 mM glyceraldehyde) to a 96-well microplate (N = 2).
  3. Add Bradford dye reagent to a 25 mL reagent reservoir using the 10 mL serological pipette.
*Note: Bradford dye reagent should be preserved at 4 °C. However, we recommend it to be kept at 22–28 °C for 30–60 min before being added into BSA in Solution D and the cell lysates.*
  4. Transfer 200 μL of Bradford dye reagent using the Nichipet 7000 to the BSA in Solution D and cell lysate in the 96-well microplates.
  5. After 5 and 10 min, measure absorbance (595 nm) using the iMark microplate reader.
- **PVDF membrane and filter papers incubated in methanol and/or PBS(-)**
  1. Cut a PVDF membrane into 9 cm × 12 cm sections and incubate in methanol for 1 min at room temperature.
*Note: This operation should be performed in a fume hood to avoid exposure to methanol.*
  2. Submerge the PVDF membrane sections in PBS(-) at room temperature.
  3. Submerge also three filter papers (9 cm × 12 cm) in PBS(-) at room temperature.
  4. Incubate both the PVDF membrane sections and three filter papers in the PBS(-) for 1 h at room temperature.
*Note: Because the PVDF membrane may be hydrophobic after incubation in methanol, excess methanol should be removed using the three filter papers, and the membrane sections should be sufficiently submerged in the PBS(-).*
- **PVDF membrane and filter papers prepared for slot blot apparatus**
  1. Set the sealing gasket onto the vacuum manifold (Figure 1).
  2. Set the gasket support plate onto the vacuum manifold (Figure 1).
  3. Set each filter paper (a total of three) onto the gasket support plate (Figures 1, 2).
  4. Set the PVDF membrane onto the filter papers (Figure 2).
  5. Incubate both the PVDF membrane and filter papers in PBS(-) for 1 h at room temperature.
  6. Fix the sample template with attached sealing screw to the sealing gasket and tighten the four screws (Figures 1–3).
*Note: Any air between the PVDF membranes and filter papers should be removed. Do not dry the PVDF membrane before adding the PBS(-) in section G. If section G cannot be performed before the PVDF membrane is dried, a little PBS(-) may be added to it. Because the PVDF membrane may be hydrophobic after incubation in methanol, excess methanol should be removed using the three filter papers, and the membrane sections should be sufficiently submerged in the PBS(-). Ensure that the four screws are tightened appropriately.*
    **Figure 2.**
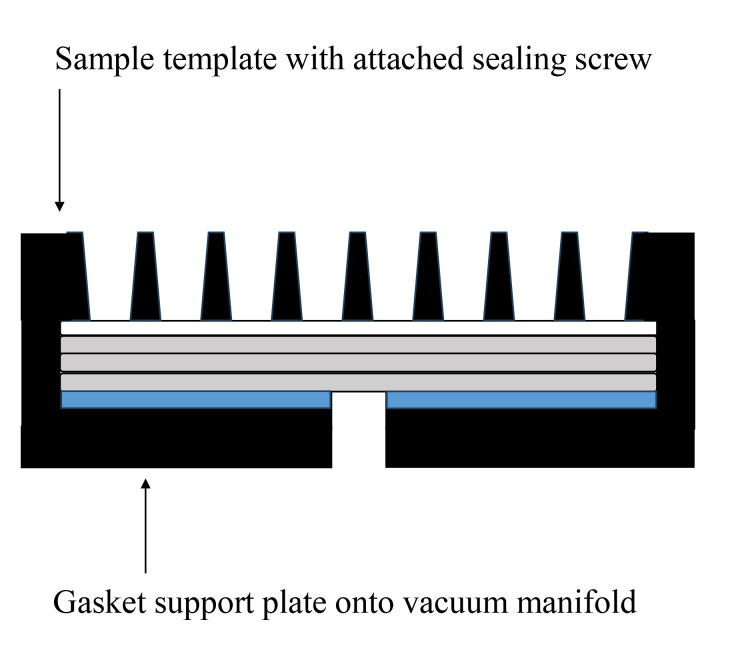
PVDF membranes and filter papers set onto slot blot apparatus. The white closed square represents the PVDF membrane, the gray closed squares represent the filter papers, and the blue closed square represents the sealing gasket.
    **Figure 3.**
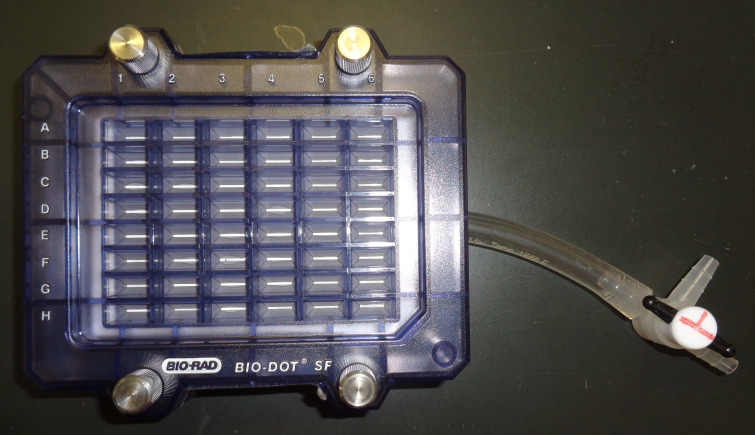
Slot blot apparatus with 48 lanes for PVDF membrane with sample template attached using sealing screws
- **Preparation of sample solution for slot blot**
  1. For the absolute quantification of the intracellular content of GA-AGEs in C2C12 [14], we selected samples containing 2 μg of protein.
*Note: To prepare the sample solution against the PVDF lanes for both “Primary antibody” and “Neutralized primary antibody,” the samples were prepared in duplicate.*
  2. Calculate the volume of the 2 μg of protein samples and move to a 1.5 mL microcentrifuge tube. Consequently, the samples with lower protein concentrations were collected in a greater volume (maximum volume of Solution D in all samples).
  3. Calculate the volume of Solution D to be added to the other samples to reach the maximum volume.
*Note: If the volume of one sample (2 μg of protein, the lowest protein concentration) was 10 μL and that of the other samples (2 μg of protein each) was 1, 3, 5, and 7 μL, Solution D was added to a final volume of 10 μL (including the cell lysates). The 1–10 μL Gilson PIPETMAN should preferably be used.*
  4. After Solution D is added to the cell lysates, incubate for 5 min at room temperature.
  5. Add PBS(-) to the cell lysates to a final volume of 200 μL for the slot blot analysis.
*Note: The 50–200-μL Gilson PIPETMAN should preferably be used.*
- **Preparation of standard GA-AGEs-BSA solution for slot blot**
  1. We prepared 2 μL of GA-AGEs-BSA solution (0, 0.5, 1.5, 5, 15, 30, and 50 ng/μL, see Recipe 9) in a 1.5 mL microcentrifuge tube.
*Note: To prepare the standard GA-AGEs-BSA solution against the PVDF lanes for both “Primary antibody” and “Neutralized primary antibody,” the samples were prepared in duplicate. The 1–10 μL Gilson PIPETMAN should preferably be used.*
  2. Add Solution D against the GA-AGEs-BSA solution.
*Note: If the volume of the sample solution is 10 μL, the same volume of Solution D must be added against the GA-AGEs-BSA solution.*
  3. After Solution D is added to the cell lysates, incubate for 5 min at room temperature.
  4. Add PBS(-) to the cell lysates up to a final total volume of 200 μL for the slot blot analysis.
*Note: The 50–200 μL Gilson PIPETMAN should preferably be used.*
- **Preparation of HRP-conjugated marker solution for slot blot**
  1. We prepared 3 μL of the HRP-conjugated marker solution (Recipe 10) in a 1.5 mL microcentrifuge tube.
*Note: To prepare the HRP-conjugated marker solution against the PVDF lanes for both “Primary antibody” and “Neutralized primary antibody,” the samples must be prepared in duplicate. The 1–10 μL Gilson PIPETMAN should preferably be used.*
  2. Add Solution D against the HRP-conjugated marker solution.
*Note: If the volume of the sample solution is 10 μL, the same volume of Solution D is added against the HRP-conjugated molecular weight marker solution.*
  3. After Solution D is added to the HRP-conjugated marker solution, incubate for 5 min at room temperature.
  4. Add PBS(-) to the cell lysates up to a final volume of 200 μL for the slot blot analysis.
*Note: The 50–200 μL Gilson PIPETMAN should be used.*
- **Selecting lanes for sample, GA-AGEs-BSA, and HRP-conjugated marker solution**
  1. The selection of lanes for the sample, GA-AGEs-BSA, and HRP-conjugated marker solution is presented in Figure 4.
*Note: The addition of samples onto the PVDF membrane is duplicated (N = 2) for both “Primary antibody” and “Neutralized primary antibody” [14]. However, the method by which one sample is applied onto each PVDF membrane (N = 1) can be performed [10,12].*
    **Figure 4.**
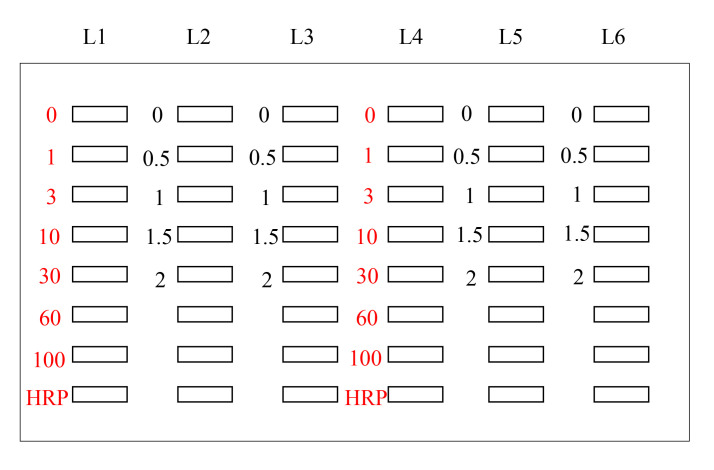
Setup for the application of standard GA-AGEs-BSA, HRP-conjugated marker, and sample solutions onto the PVDF membrane (setup explained in depth in Takata et al. [14]). White open squares indicate slot lanes. L1, L4: 0, 1, 3, 10, 30, 60, and 100 ng of GA-AGEs-BSA aliquots and HRP-conjugated marker solution. L2, L3, L5, and L6: C2C12 cell lysate samples treated with 0, 0.5, 1, 1.5, and 2 mM glyceraldehyde for 24 h. (This figure was reproduced from Takata [8]; copyright belongs to Takata T.)
- **PBS(-) wash of the PVDF membrane in slot blot apparatus**
  1. Add 100 μL of PBS(-) to the slot blot apparatus without water aspiration.
*Note: The Nichipet 7000 was used.*
- **Sample, GA-AGEs-BSA, and HRP-conjugated marker solution applied onto the PVDF membrane and removed with water aspiration**
  1. Apply 200 μL of sample, GA-AGEs-BSA, and HRP-conjugated marker solutions.
*Note: The 50–200 μL Gilson PIPETMAN should preferably be used. This operation should be performed with the water aspirator (Figure 5).*
  2. Place the slot blot apparatus on a stand near the water aspirator.
*Note: Any stand may be used for the experiment, as long as it has a height of 25–30 cm (Figure 6).*
  3. Connect a tube from the flow valve to the water aspirator (Figure 6).
  4. Vacuum the sample, GA-AGEs-BSA, and HRP-conjugated marker solutions with water aspiration while opening one valve for air (Figure 7A).
  5. For complete sample addition, we recommend vacuuming with water aspiration while the valve is closed for air (3–5 s) (Figure 7B).
*Note: Although water aspiration pressure is not specified, this can be estimated. Vacuuming with water aspiration was performed at the Kanazawa Medical University, where the water supply is collected in a water tank and redistributed between laboratories. Here, the water pressure is consistent with that of a typical household (0.15–0.74 MPa) or corporate water supply system in Uchinada (0.20–0.23 MPa), according to data from the Ministry of Health, Labour, and Welfare in Japan. Therefore, the water supply system at Kanazawa Medical University has been adjusted to a pressure of 0.20–0.23 MPa.*
    **Figure 5.**
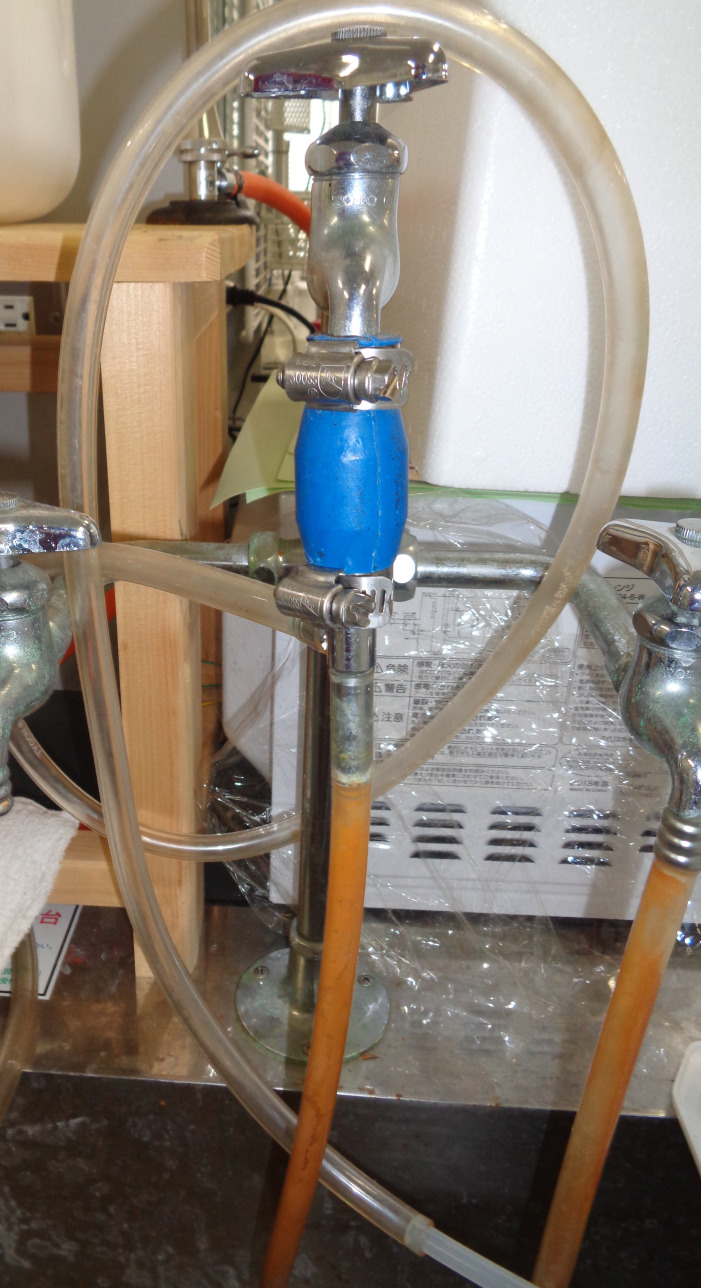
Water aspirator (AS ONE) in the laboratory at Kanazawa Medical University
    **Figure 6.**
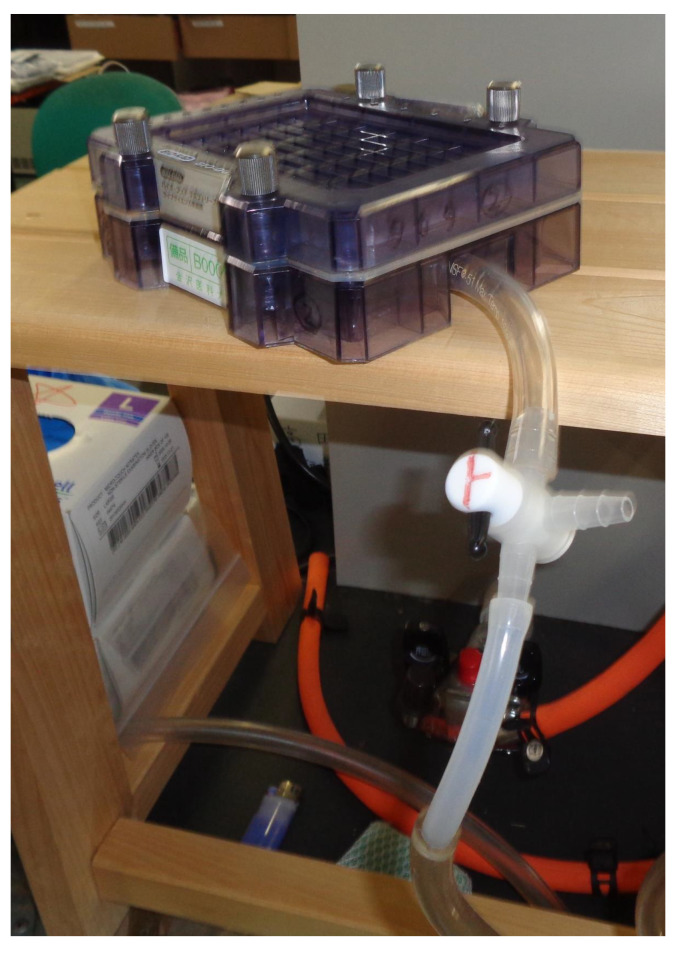
Slot blot apparatus on a stand; the large tube runs between the flow valve and water aspirator
    **Figure 7.**
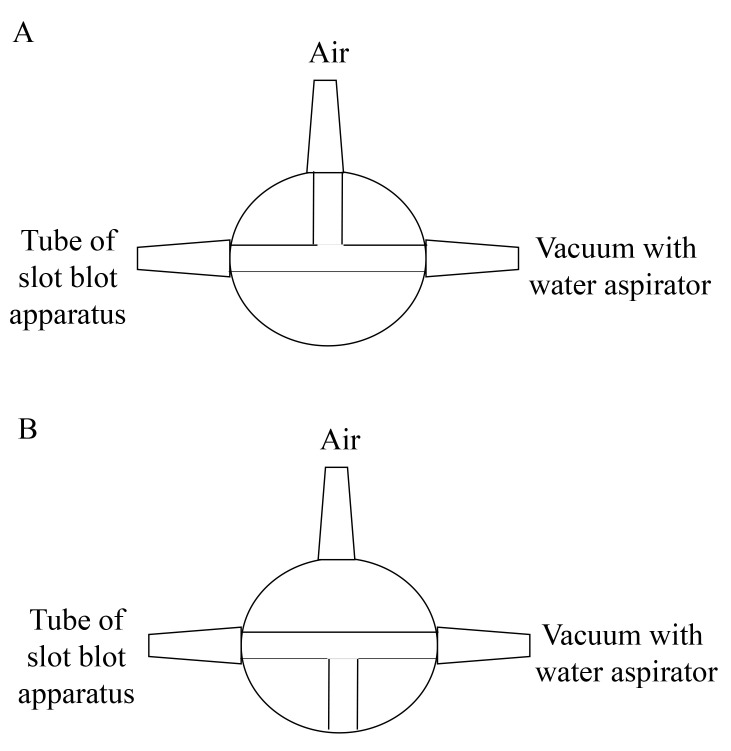
Valve between slot blot apparatus and vacuum with water aspirator. A. The vacuuming with one valve opened against air. B. The vacuuming with the valve closed against air (This figure was reproduced from Takata et al. [9]. Copyright belongs to Takata T., Masauji T., and Motoo Y., who permitted the use of this image.)
- **PBS(-) wash of the PVDF membrane in slot blot apparatus following section L**
  1. Add 200 μL of PBS(-) to the slot blot apparatus.
*Note: The Nichipet 7000 was used.*
  2. Vacuum PBS(-) with water aspiration while opening one valve for air (Figure 7A).
  3. For complete sample addition, we recommend vacuuming with water aspiration while the valve is closed for air (3–5 s) (Figure 7B).
- **Milli-Q water wash of the PVDF membrane**
  1. Remove the PVDF membrane from the slot blot apparatus.
  2. Incubate the PVDF membrane in Milli-Q water in a disposable polypropylene tray (AS ONE) for 1 min at room temperature.
- **PVDF membrane dissection and incubation in 5% SM-PBS-T**
  1. Cut the PVDF membrane into smaller sections (9 cm × 6 cm).
  2. For blocking, incubate each PVDF membrane section in 5% SM-PBS-T (15 mL) for 30 min at room temperature (see Recipe 12).
*Note: Each PVDF section was placed into a disposable polypropylene tray and processed using the seesaw shaker (BIO CRAFT).*
- **PVDF membrane incubation in primary and neutralized primary antibody solutions**
  1. Make two sample packs using the Hybri-Bag (hard type).
*Note: The pack should contain the PVDF membrane section (size: 9 cm × 6 cm) and ~5 mL of solution.*
  2. Wash each PVDF membrane with 0.5% SM-PBS-T (10 mL) for 5 min at room temperature. Perform this operation twice (see Recipe 13).
*Note: Each PVDF membrane section is placed into a disposable polypropylene tray and incubated on the seesaw shaker (BIO CRAFT) in a refrigerator or cooled room.*
  3. Pack one PVDF membrane with primary antibody solution (see Recipe 14) and another with neutralized primary antibody solution (see Recipe 15).
*Note: The primary and neutralized antibody solutions must be prepared and incubated on the seesaw shaker for 1 h at room temperature before this step.*
  4. Incubate both packs overnight at 4 °C.
*Note: Incubation on the seesaw shaker (BIO CRAFT) could be performed in a refrigerator or a cooled room.*
- **PVDF membrane incubation in secondary antibody solution**
  1. Remove each PVDF membrane from the primary or neutralized primary antibody solution.
  2. Incubate each PVDF membrane in 0.5% SM-PBS-T (10 mL) for 10 min at room temperature, three times.
*Note: Each PVDF membrane section is placed into a disposable polypropylene tray and incubated on the seesaw shaker (BIO CRAFT) in a refrigerator or cooled room.*
  3. Make two sample packs using the Hybri-Bag.
*Note: The pack should contain the PVDF membrane section (9 cm × 6 cm) and ~5 mL of solution.*
  4. Pack each PVDF membrane with a secondary antibody solution (see Recipe 16).
  5. Incubate each PVDF membrane for 1 h at room temperature.
*Note: The incubation on the seesaw shaker (BIO CRAFT) should be performed.*
- **PVDF membrane incubation in PBS-T and PBS(-)**
  1. Remove each PVDF membrane from the secondary antibody solution.
  2. Incubate each PVDF membrane in PBS-T (15 mL) twice for 10 min at room temperature.
*Note: Each PVDF is placed into a disposable polypropylene tray and processed using the seesaw shaker (BIO CRAFT).*
**Pause point:** The PVDF membranes could be incubated in PBS-T on the seesaw shaker for 3–12 h at room temperature before the next step.
  3. Incubate each PVDF membrane section in PBS(-) (15 mL) at room temperature.
*Note: Incubation is generally performed for 10 min before section S.*
- **Chemiluminescence imaging of bands on PVDF membrane (see General note 2)**
  1. Make two sample packs using the Hybri-Bag.
  2. Take 500 μL of reagents A and B of an ImmunoStar LD kit on the commercial sheet (e.g., polyvinylidene chloride sheet). Perform this operation twice.
  3. Mix Reagents A and B.
  4. Smear each PVDF membrane with the reagent mixture for 10 s at room temperature.
*Note: The volume of the mixture of reagents A and B in the ImmunoStar LD kit is 1,000 μL. The area of the PVDF membrane is approximately 27 cm^2^ (9 × 3 cm). The reagent solution density is 1,000 μL/27 cm^2^ = 37.0 μL/cm^2^.*
  5. After removing the reagent mixture, place each PVDF membrane section on the sheet for 30 s at room temperature.
*Note: This procedure processes two PVDF membranes simultaneously.*
  6. Pack each PVDF membrane and analyze using the Fusion FX imager.
  7. Obtain the images of the two PVDF membranes in the standard mode.
*Note: We recommend an exposure time of 0.5–30 s. Two PVDF membranes should be exposed at the same time (e.g., 10, 15, 20, and 30 s). We recommend that images be obtained in standard, high, or ultra mode (if it is difficult to obtain images).*

## Data analysis

**Calculation of protein concentration in cell lysates**

**Figure 8.**
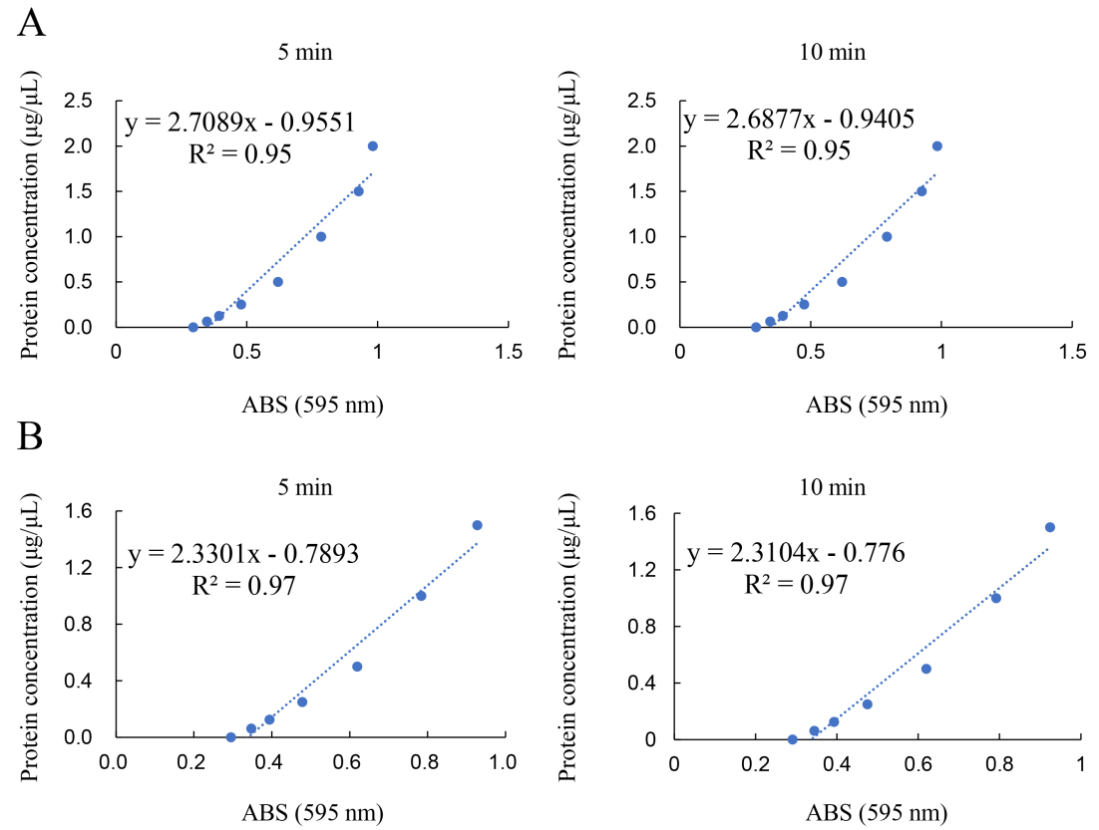
Standard curve of BSA in Solution D. The average concentration of the standard BSA solution was calculated from duplicate measurements. The examination for this example data was performed on June 29, 2022. ABS; absorbance. R^2^; correlation coefficient. A. The range was from 0 to 2.0 μg/μL. Left: Measurement of absorbance for Bradford dye treatment for 5 min. Right: Measurement of absorbance for Bradford dye treatment for 10 min. B. The range was from 0 to 1.5 μg/μL. Left: Measurement of absorbance for Bradford dye treatment for 5 min. Right: Measurement of absorbance for Bradford dye treatment for 10 min.

A standard curve was drawn based on the absorbance data measured using the iMark microplate reader (595 nm). The average concentration of the standard BSA solution was calculated from duplicate measurements (Figure 8). Similarly, the average value for the samples was calculated from duplicate measurements. The sample protein concentration was averaged from two standard curves corresponding to the 5- and 10-min Bradford dye treatments. *Note: If the absorbance of all samples is less than one of 1.5 μg/μL, the range from 0 to 1.5 μg/μL can be selected to draw the standard curve, whose value of correlation coefficient (R^2^) is high grade.*

**Detection of chemiluminescence bands on the PVDF membrane**

**Figure 9.**
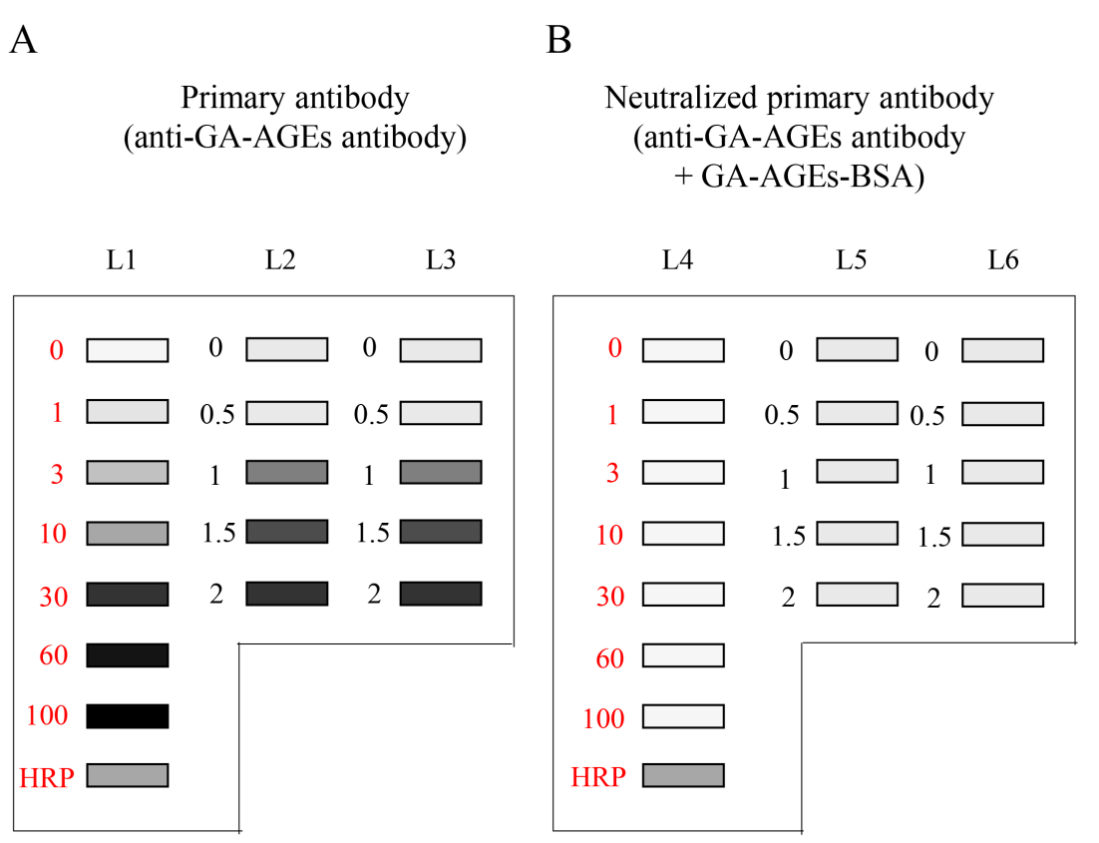
Chemiluminescence detection of standard GA-AGEs-BSA aliquots, HRP-conjugated marker solution, and sample solution on PVDF membranes. Both membrane sides were simultaneously exposed using the Fusion FX imager. A. (L1–L3): Anti-GA-AGE antibody was probed onto the PVDF membrane. B. (L4–L6): Neutralized anti-GA-AGE antibody was probed onto the PVDF membrane. Closed gray and black squares indicate bands on the PVDF membrane. L1, L4: 0, 1, 3, 10, 30, 60, and 100 ng of GA-AGEs-BSA aliquots and HRP-conjugated marker solution. L2, L3, L5, L6: Cell lysate samples of C2C12 cells treated with 0, 0.5, 1, 1.5, and 2 mM glyceraldehyde for 24 h. (This figure is from Takata [8]; copyright belongs to Dr. Takata.)

To detect the chemiluminescence band image, images were exposed with the chemiluminescence mode of Fusion FX equipment, and the image was reflected in the general personal computer that contained Fusion FX software (version 17.03). The example chemiluminescence bands image of GA-AGEs-BSA and intracellular GA-AGEs in cell lysates on PVDF membranes are published in Supplementary Figure 1a in reference [14]. The model images are described in Figure 9.

## Supplementary information

The HRP-conjugated marker solution was used as a control. The corrected luminance value (arbitrary unit, AU) was calculated as follows: Calibration-corrected luminance value = (HRP Left – Blank Left) / (HRP Right – Blank Right).

*Note: We performed three independent experiments. Statistical analysis of one-way ANOVA and Tukey tests were used to quantify intracellular GA-AGE levels [14].*

## Validation of protocol

This protocol or parts of it have been used and validated in Takata et al. [14] (DOI: 10.1186/s13098-020-00561-z).

The chemiluminescence bands on PVDF membranes are shown in Supplementary Figure 1a of Takata et al. [14].

The detection of bands of standard GA-AGEs-BSA and intracellular GA-AGE using the novel slot blot approach was validated in various cells under the same or similar protocols [10–13,15–17].

## General notes and troubleshooting

**General notes**

1. Measurement of protein concentration in cell lysates
  We discourage the use of commercial standard BSA because it contains various compounds that may affect absorbance. Instead, we recommend that BSA powder be dissolved in Solution D.
*Note: This method can be applied to tissue lysates [15,16].*
2. Chemiluminescence imaging of bands on PVDF membranes
  Although we used the Fusion FX imager and its software [14], other imaging equipment may be used [10,12,17].
  For example, a LAS4000 system (GE Healthcare) was used when this novel slot blot approach was developed in 2017 [10].
3. Limitation
  The volume and mass of samples should be less than 500 μL and 10 μg of protein, respectively.

**Troubleshooting**

Problem 1: Bands are invisible in chemiluminescence images (Case 1).

Possible cause: Proteins in cell lysates are unable to be probed onto the PVDF membrane because it was completely dried before the GA-AGEs-BSA, HRP-conjugated marker, and sample solution were added to it.

Solution: GA-AGEs-BSA, HRP-conjugated marker, and sample solutions should be rapidly added onto the PVDF membrane after the PBS(-) wash.

Problem 2: Bands are invisible in chemiluminescence images (Case 2).

Possible cause: Proteins in cell lysates are unable to be probed onto the PVDF membrane because Solution D contains Triton-X.

Solution: Confirm the composition of Solution D, which should not contain Triton-X.

Problem 3: Excessive background noise in PVDF membrane detection.

Possible cause: The volume of Solution D added onto the PVDF membrane may be excessive.

Solution: We recommend using 5–15 μL of Solution D, while cell lysates should be prepared with 150–200 μL of Solution D.
